# Genomic Investigations Unmask *Mycoplasma amphoriforme*, a New Respiratory Pathogen

**DOI:** 10.1093/cid/ciu820

**Published:** 2014-10-23

**Authors:** Stephen H. Gillespie, Clare L. Ling, Katarina Oravcova, Miguel Pinheiro, Louise Wells, Josephine M. Bryant, Timothy D. McHugh, Cecile Bébéar, David Webster, Simon R. Harris, Helena M. B. Seth-Smith, Nicholas R. Thomson

**Affiliations:** 1 School of Medicine, University of St Andrews, United Kingdom; 2 Shoklo Malaria Research Unit, Mahidol-Oxford Tropical Medicine Research Unit, Faculty of Tropical Medicine, Mahidol University, Mae Sot, Thailand; 3 University College London Centre for Clinical Microbiology, Royal Free Campus, and; 4 Wellcome Trust Sanger Institute, Hinxton, United Kingdom; 5 University of Bordeaux, France; 6 Department of Immunology, Royal Free Hospital NHS Trust, London, United Kingdom

**Keywords:** *Mycoplasma amphoriforme*, whole genome sequencing, respiratory infection, infection control, primary antibody deficiency

## Abstract

***Background.*** *Mycoplasma amphoriforme* has been associated with infection in patients with primary antibody deficiency (PAD). Little is known about the natural history of infection with this organism and its ability to be transmitted in the community.

***Methods.*** The bacterial load was estimated in sequential sputum samples from 9 patients by quantitative polymerase chain reaction. The genomes of all available isolates, originating from patients in the United Kingdom, France, and Tunisia, were sequenced along with the type strain. Genomic data were assembled and annotated, and a high-resolution phylogenetic tree was constructed.

***Results.*** By using high-resolution whole-genome sequencing (WGS) data, we show that patients can be chronically infected with *M. amphoriforme* manifesting as a relapsing-remitting bacterial load, interspersed by periods when the organism is undetectable. Importantly, we demonstrate transmission of strains within a clinical environment. Antibiotic resistance mutations accumulate in isolates taken from patients who received multiple courses of antibiotics.

***Conclusions.*** *Mycoplasma amphoriforme* isolates form a closely related species responsible for a chronic relapsing and remitting infection in PAD patients in the United Kingdom and from immunocompetent patients in other countries. We provide strong evidence of transmission between patients attending the same clinic, suggesting that screening and isolation may be necessary for susceptible patients. This work demonstrates the critical role that WGS can play in rapidly unraveling the biology of a novel pathogen.

## Abstract (precis)

The results of high-resolution whole-genome sequencing data provide compelling evidence that M*ycoplasma amphoriforme* produces chronic relapsing infection and, importantly, is transmitted in a hospital environment.

Lower respiratory tract infection (LRTI) is an important cause of morbidity and mortality in all age groups, especially in immunocompromised patients. An etiological agent is only found in approximately 70% of cases despite intensive investigation [1]. *Mycoplasma amphoriforme* was first isolated from a patient with X-linked agammaglobulinemia with chronic bronchitis in 1999. The patient was expectorating a large volume of sputum that tested negative for all other recognized lower respiratory tract pathogens [2]. The organism was identified as a novel bacterial species and given the name *M. amphoriforme* (MAM) [2]. Based on 16S ribosomal RNA (rRNA) gene sequencing, the closest species is *Mycoplasma testudinis* [2]. MAM grows poorly on standard mycoplasma media and lacks the appearance of other *Mycoplasma* species. Only a limited number of strains have been isolated worldwide, and the majority of isolates are from patients with primary antibody deficiency (PAD) attending a single specialist clinic in London, with additional isolates from immunocompetent patients in Denmark, France, and Tunisia [3].

Considering the burden of disease from LRTIs on human health and the recent association of MAM with LRTI in immunocompetent patients [1, 4], it is important to understand the dynamics of chronic infection with this organism and to determine whether it is being transmitted among vulnerable patients and how these strains relate to other isolated strains. Using the available clinical case notes and whole-genome sequencing (WGS), we provide evidence for multiple strains circulating internationally, chronic relapsing respiratory infection, and, crucially, patient-to-patient transmission within a hospital environment.

## METHODS

### Patients, Samples, and Ethical Approval

The Ethics Committee of the Royal Free London National Health Service Foundation Trust (RFL) approved these studies. Sputum samples were collected from a total of 88 adult patients with PAD attending the PAD Clinic at the RFL and tested for MAM using *Mycoplasma* culture, a 16S rRNA gene MAM-specific polymerase chain reaction (PCR) (16S PCR), and a uracil DNA glycosylase MAM-specific quantitative PCR (*udg* quantitative polymerase chain reaction [qPCR]) [4]. A total of 19 sequential isolates from 9 of the 17 MAM positive patients were available for WGS. Additionally three patients from three patients reported previously provided by the University of Bordeaux (Supplementary Table 1) [3]. The clinical information was reviewed for evidence of symptoms associated with LRTI.

### Extraction of DNA

Extraction of DNA from sputum samples was performed using a Chelex-based method as previously described [4]. DNA for WGS was extracted using the Wizard Genomic DNA extraction kit (Promega, Southampton, UK) following the manufacturer's instructions using the protocol for gram-negative bacteria, and amplified using the illustra Genomiphi V2kit (GE Healthcare), according to the manufacturer's instructions.

### MAM-Specific PCR

The MAM-specific conventional 16S PCR was performed prospectively, and the real-time qPCR targeting *udg* was performed retrospectively on DNA extracts from PAD patient samples as described previously [4].

### WGS: Reference Genome

The genome of the reference MAM strain A39 was Sanger sequenced to a depth of 8 times coverage using cloning vector p0TWI2 with a selection of insert sizes (2–3 kb, 3–4 kb, and 4–5 kb) using dye terminator chemistry on ABI3700 automated sequencers (Life Technologies Ltd, Paisley, UK). Repetitive regions of the genome were spanned and the assembly manually was finished using long-range PCR and pair read information. The genome was annotated as described previously, using Artemis [5–7]. The genome was sequenced to a level classified as “finished.” The MAM genomic DNA sequence was compared against the European Molecular Biology Laboratory (EMBL) prokaryote database using BLASTN and BLASTX [8]. Transfer RNAs were predicted by tRNAscan-SE [9]. Potential coding sequences were predicted using GLIMMER [10] and the results were combined and checked manually. The predicted protein sequences were searched against a nonredundant protein database using WUBLASTP and FASTA. The complete 6-frame translation was used to search PROSITE [11], and the predicted proteins were compared against the Pfam database of protein domain [12] using hidden Markov models and the Conserved Domain Search tool against the Conserved Domain Database from the National Center for Biotechnology Information [13]. The results of these analyses were compiled using Artemis and used for a manual gene-by-gene annotation of the sequence and predicted proteins. Annotation was based, wherever possible, on characterized proteins or genes. Repeat sequences were identified using the Dotter program [14] and manually.

### WGS: Clinical Isolates

WGS was performed on single isolates on a MiSeq Instrument (Illumina, San Diego, California). A 300-cycle MiSeq Reagent Kit version 2 was used to generate 75-bp paired-end reads. The reads obtained for each isolate were mapped against the MAM reference strain A39 with SMALT (Available at: http://www.sanger.ac.uk/resources/software/smalt/). Single-nucleotide polymorphisms (SNPs) were identified using SamTools Mpileup [15] and bcftools, and filtered as previously described [16].

### Accession Numbers

The raw sequence data are available under the accession number ERP000340. The sequence and annotation for MAM A39 have been deposited with EMBL (accession number HG937516).

### Phylogenetic Analysis

Regions with high-SNP density or SNPs within repetitive regions were excluded from the phylogenetic analysis as described previously [17]. Maximum likelihood phylogenetic trees were constructed with randomized axelerated maximum likelihood using a generalised time reversible evolutionary model and a γ-correction for among-site rate variation. Support for relationships in the maximum-likelihood phylogenetic tree was assessed by running 100 bootstrap replicates.

### Detecting Minority Variants

Minority variants were extracted from the mapping data using stringent filters to distinguish true variants from sequencing or mapping errors. A variant was only counted if confirmed by at least 4 reads with at least 2 reads on each strand, and a base and mapping quality of 50 and 30, respectively. The variants required strand bias *P* value of at least .05, depth of coverage within a normal range (± 50% of the average), and a distance of at least 200 bp from another variant. As depth of coverage varies across the genome, we corrected each count of a minority variant by dividing it by the depth. For example, for a particular position with a depth of coverage of 100 reads, we recorded 0.01. We summed these values to produce a minority variant score for each sample, with higher scores indicating higher diversity in the sample.

## RESULTS

### Patients, Longitudinal Samples, and Bacterial Load

**Figure 1.**
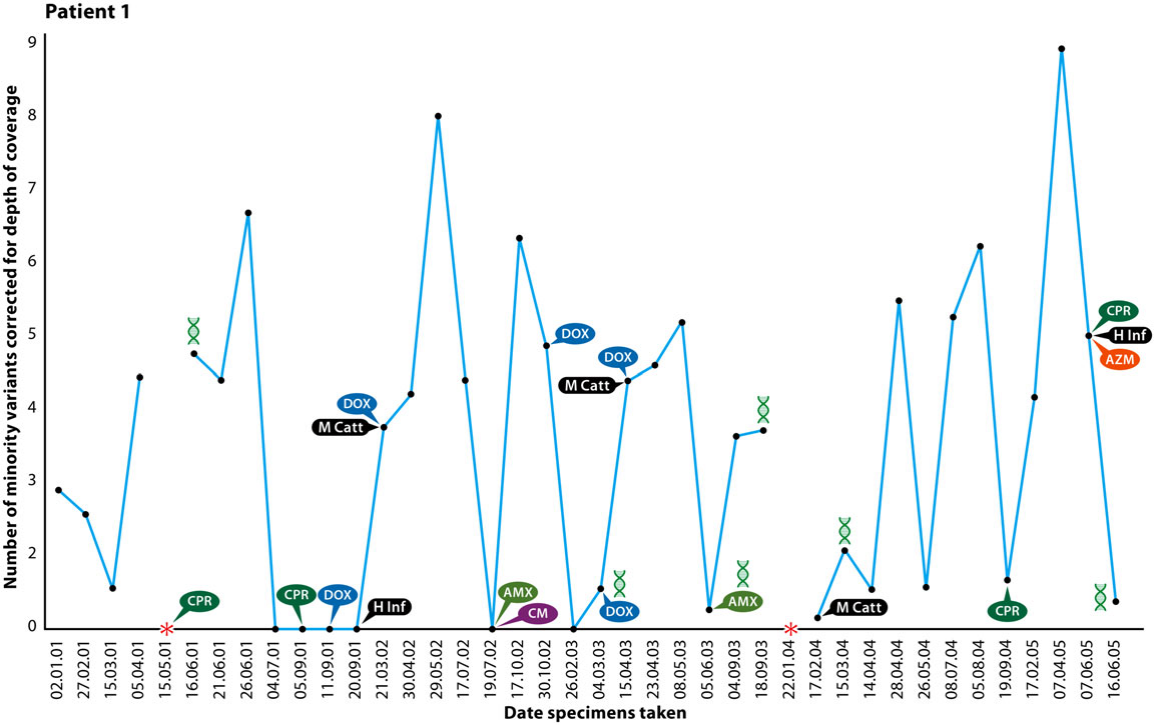
Natural history of a patient with *Mycoplasma amphoriforme* infection (patient 1). Colony-forming units are estimated using the *udg* quantitative polymerase chain reaction (PCR). The symbols represent the bacteria isolated and the antibiotic treatment used. *udg PCR was not performed, but the patient was positive by culture or 16S ribosomal RNA PCR. Abbreviations: AMX, amoxicillin; AZM, azithromycin; CM, clarithromycin; CPR, ciprofloxacin; DOX, doxycycline; H Inf, *Haemophilus influenzae*; M Catt, *M. cattarhalis*.

All of the patients had evidence of chronic productive cough and some experienced high sputum volumes. The duration over which samples were collected ranged from 2 to 5 years with the number of recurrent episodes of MAM-associated LRTIs ranging between 4 and 11 episodes for which clinical data are available. It is notable that half of the patients had evidence of obstructive airway disease while infected with MAM. Of the 94 serially collected samples available for laboratory investigation from 9 patients, 53 of 92 (57.6%) were found to be positive for MAM by culture, 85 of 92 (92.4%) were positive by 16S PCR, and 81 of 89 (91%) were positive by qPCR. No other recognized LRTI bacterial pathogen was identified in 51 of the 64 samples for which routine sputum culture results were available. Of those samples positive for MAM where another LRTI pathogen was cultured, 3 samples contained *Haemophilus influenzae*, 6 *Streptococcus pneumoniae*, and 3 *Moraxella catarrhalis* (Figure 1). A summary of the findings for all MAM-positive samples is given in Supplementary Table 1.

Bacterial load data obtained by qPCR indicate prolonged infection (Figure 1 and Supplementary Figure 1). We use patient 1, from whom the original isolate and now the type strain, A39, was isolated [18], as an exemplar of the observed course of infection. This patient experienced a chronic course of productive purulent or mucopurulent sputum for >4 years with a consistently high MAM bacterial load (Figure 1). Of the 40 samples taken between 2001 and 2005 from patient 1 (Figure 1), MAM was detected by PCR on all but 5 occasions; MAM culture was positive for 22 specimens, negative for 14, contaminated for 4, and not performed for 1. The only other recognized bacterial pathogens identified in the MAM-positive samples from this patient were *M. catarrhalis* and *H. influenza*e (Figure 1). Patient 8 also followed a chronic course, with evidence of a productive cough at all 18 visits over 4.5 years with purulent or mucopurulent sputum. The samples were MAM culture positive on 10 occasions and all those tested by PCR were positive, with only a single isolation of *S. pneumoniae* during this period.

### Genome Sequences of Clinical Isolates

WGS has the benefit of providing the highest possible resolution data with which to differentiate closely related isolates. The A39 genome is 1.03 Mb with a 31.6% G + C content. Furthermore, whole-genome draft sequences using MiSeq were determined for 19 of the 35 cultured MAM isolates, this set being limited by isolates that failed to reculture.

By calling SNPs against our high-quality reference sequence, we constructed a maximum likelihood phylogenetic tree taking account of possible recombination [19] by using the method of Croucher et al [17]. Supplementary Figure 2 shows the regions of high SNP density identified in the MAM isolate genomes and may be representative of recombination events from bacteria beyond the studied isolates.

**Figure 2.**
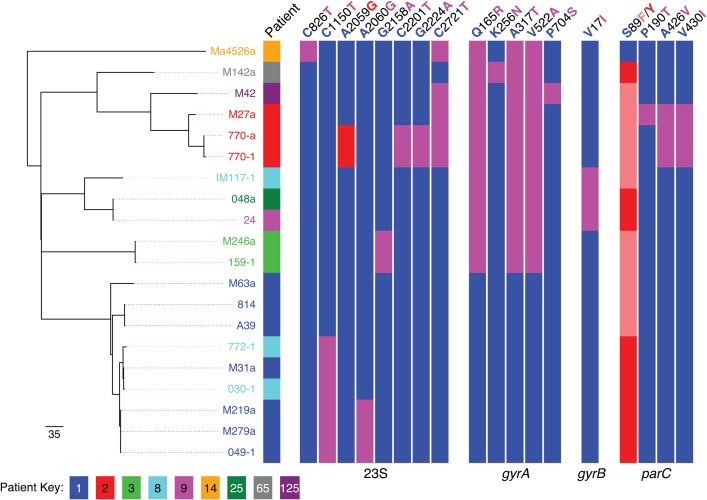
Maximum likelihood phylogenetic tree for 20 isolates of *Mycoplasma amphoriforme* from 9 patients distinguished by color and the strain designated by its code (8 from Royal Free London National Health Service Foundation Trust and 1 of 3 French/Tunisian isolates). The table was constructed with randomized axelerated maximum likelihood using a Generalised time reversible evolutionary model and a γ-correction for among-site rate variation. Single-nucleotide polymorphisms (SNPs) are noted for the 23S ribosomal RNA, and nonsynonymous SNPs for *gyrA*, *gyrB*, and *parC* genes using *M. amphoriforme* numbering. For each, a red or salmon-pink bar indicates evidence of association of the SNP with phenotypic antibiotic resistance, pink a possible association, and blue represents the ancestral, sensitive allele. Bootstrap support values for the relationships shown in the phylogeny can be found in Supplementary Figure 3.

**Figure 3.**
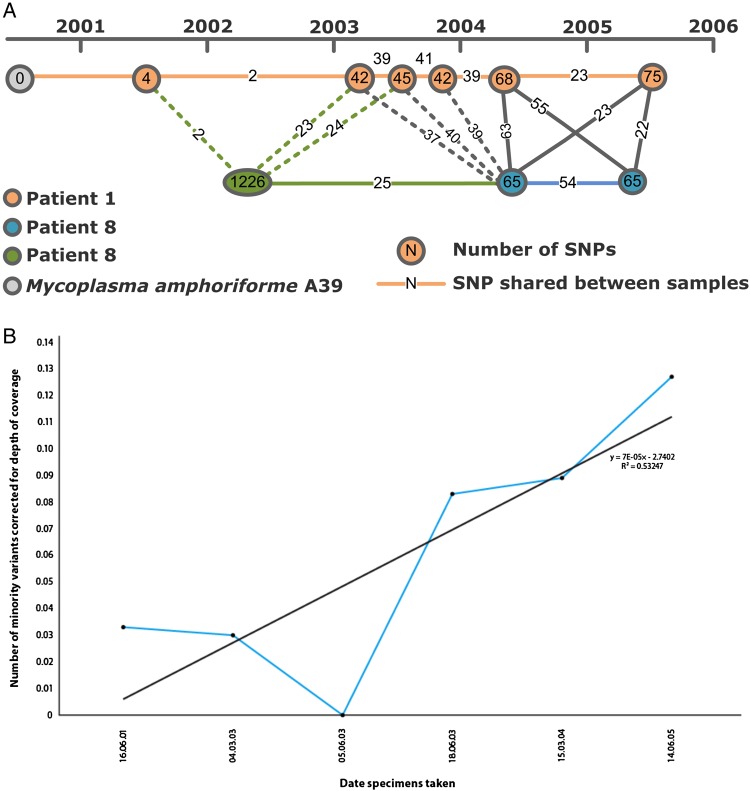
*A*, Accumulation of single-nucleotide polymorphisms (SNPs) detected in available *Mycoplasma amphoriforme* isolates within patient 1 (orange) and 8 (green or blue) from 1999 to 2006. Numbers indicate the number of SNPs the isolates have compared to the A39 type strain sequence, which was also isolated from patient 1. *B*, Measure of clinical isolate diversity detected at different sampling times for patient 1. The numbers represent the numbers of minority variants detected, corrected for depth of coverage. Minority variants were counted if they were supported by at least 4 reads, with 2 reads on each strand and a base and mapping quality of 50 and 30, respectively. There is a positive nonsignificant trend between time and number of minority variants, indicating an increase of diversity over the course of the infection (linear regression model: *r*^2^ = 0.532, *P* = .099).

The phylogenetic tree (Figure 2) shows that the sequenced MAM isolates fall into 5 main clades: 4 represented by UK isolates taken from patients attending the RFL and a fifth containing a single out-group strain (Ma4526a). Additionally, 1 French, 1 Tunisian, and 1 UK strain were highly diverse and were not included in this phylogenetic analysis (Figure 2 and Supplementary Figure 3). The overall level of sequence-based variation is low, with distances between clades ranging from 159 to 254 SNPs, and a maximum of 76 SNPs (minimum = 4) separating any pair of isolates within 1 clade. It is clear that the UK isolates from the RFL are more similar to each other than the French out-group strains. Isolates from an individual are more closely related to each other than to those from other patients . Where we have multiple longitudinal samples taken from a single patient (eg, patient 1), the phylogeny does not appear to be entirely consistent with the dates of sample collection. Some isolates that appear more basal in the tree were collected more recently (Figures 2 and 3*A*), Across the strain set, we detected 636 minority variants, and in patient 1 there was a trend toward increasing numbers of numbers of minority variants over time (Figure 3*B*).

### Cross-infection and Evolution

There were several different patterns of infection (Figure 2): patient 3 presented twice with almost identical isolates from the same lineage between 2002 and 2003; patients 125 and 65 each only contributed single samples that could be sequenced, which are closely related to but distinct from the UK RFL isolates. The comparative SNP analysis of MAM isolates from patient 8 indicates a probable transmission event. The first sequenced isolate from patient 8 (IM117-1; Figure 2) collected in 2002 falls within clade 2, but is distinct from the other members of this clade that is, a distinct strain. The sequences of the subsequent isolates (O30-1 and 772-1) collected in 2004 and 2005 fall within the diversity of the isolates from patient 1 (Figures 2 and 3*A*). The collection dates of the specimens indicate that patient 1 and patient 8 attended the outpatient clinic on the same day on 2 occasions. The 2003 isolates from patient 1 share ≥37 SNPs, with the latter isolate from patient 8; the highest number of shared SNPs between the isolates of these 2 patients is found in early 2004, suggesting transmission around this date (Figure 3*A*).

### Antibiotic Resistance

From sequence analysis, there were no whole-gene acquisitions within the course of any of the UK PAD patient infections, providing no evidence of resistance acquisition by horizontal gene transfer (eg, *tetM*). A total of 64 SNPs were identified in all isolates in *gyr*A, 11 of which were nonsynonymous, but none were found in the quinolone-resistance determining region (QRDR). Among 43 SNPs detected in *gyr*B, there were 3 nonsynonymous amino acid changes at positions V17I, P389Q, and V620A. A total of 23 nonsynonymous SNPs were identified in the *par*C gene, 1 at position 89 (position 80 in *Escherichia coli* numbering) found within the QRDR. The 3 isolates from France and Tunisia had a serine residue at the position 89 (associated with a susceptible phenotype), whereas all UK isolates, including the reference isolate A39, had either F or Y substitutions, implying that the UK isolates were quinolone resistant. A total of 22 mutations were detected in the 23S rRNA gene, 1 of which was located within a region associated previously with macrolide resistance. Two strains from patient 2 (both isolated in 2001) both carry an A2059G substitution in the 2 latest isolates of this series. In vitro susceptibility testing of this isolate showed that this strain had a minimum inhibitory concentration of 0.2 µg/L and 1.5 µg/L for doxycycline and ciprofloxacin, respectively.

## DISCUSSION

There is, as yet, little clinical, pathological, molecular, or genomic information about *M. amphoriforme* [4], and this study significantly expands our understanding. Patients with PAD are especially susceptible to infections of the respiratory tract, otitis media, and sinusitis [20]. Pneumonia is a common reason for presentation; it may recur on multiple occasions [21] and can result in death [22]. Chronic infection is common and often complicated by bronchiectasis [22], an important prognostic indicator [23]. Patients with PAD often are commonly infected with *S. pneumoniae*, *H. influenzae*, *Staphylococcus aureus*, and *Pseudomonas* species [20]. In these patients, joint, urogenital, and respiratory tract infections may be caused by *Mycoplasma* species. *Ureaplasma urealyticum*, *Mycoplasma orale*, and *Mycoplasma pneumoniae* were identified during episodes of respiratory infection in 18 of 23 patients [24], and *Mycoplasma hominis* has also been reported in an immunocompetent patient [25].

The genome of MAM has a similar G + C content to other *Mycoplasma* species, and a detailed gene-by-gene analysis of the type strain A39 will be published subsequently. The isolates from patient 1 are sufficiently related to each other to indicate chronic infection with the same strain. The bacterial load varies (Figure 1 and Supplementary Figure 1), indicating a relapsing-remitting course in patients with PAD, lasting at least 1626 days (Figure 1) and associated with purulent and mucopurulent sputum. Patients were *udg* qPCR positive on all but 7 occasions (Figure 1 and Supplementary Figure 1). WGS data demonstrate that relapse was with the same strain, suggesting that intervening qPCR negatives were false negatives or below the limit of qPCR detection.

Relapsing respiratory symptoms are common in PAD patients and, in the absence of other recognized respiratory pathogens and the continued presence of symptoms, it suggests that MAM is causing bronchial inflammation. A similar relapsing course is seen in immunocompetent patients with chronic obstructive pulmonary disease where bacterial load is higher during exacerbation than during stable state [26]. Control of bacterial load is associated with a reduced risk of relapse [27]. Continuing purulent sputum and obstructive airway disease suggest ongoing airway inflammation in association with MAM. Further work is required to elucidate the relationship between this organism and changing respiratory function.

A trend for an increase in minority variants in a chronically infected individual who failed to achieve statistical significance may suggest that organisms exist as an increasingly heterogeneous mixture in chronic infection. This result should be treated with caution as it is unknown what affect whole-genome amplification may have on the error rate of the sequencing reads. In addition, we do not know how many variants may have been generated or selected for during in vitro growth of these samples. The limited number of samples and sampling depth is insufficient to capture the full diversity of isolates infecting a single person at a particular time-point. However, this observation is consistent with studies from chronic infections [28] and explains the complex relationship between phylogeny and date of isolation. In an outbreak of *S. aureus* in a neonatal care unit, the strain carried by a staff member differed by up to 27 SNPs when different colonies from a sample were sequenced independently [28], and is consistent with the poor correlation between isolation date and phylogenetic relatedness of serial samples from patients shown here.

Patients with anatomical or genetic deficits or immunocompromise are susceptible to cross-infection in the hospital environment and respiratory pathogens in a clinic setting such as *Mycobacterium tuberculosis* in human immunodeficiency virus–positive patients and *Burkholderia cepacia* in patients with cystic fibrosis [29–31]. As most *M. amphoriforme* strains were isolated at a single hospital, it was possible that this represented an unrecognized nosocomial outbreak. WGS has enabled us to answer this question unequivocally. Almost all of the patients investigated in this collection had a genetically distinct strain in their lungs, which excludes the possibility of this being a point source outbreak. The interaction between patients 1 and 8 is significant, as these patients were in the clinic on the same day on at least 2 occasions in 2002 and 2003. The strain isolated from patient 8 in 2002 is distinct from the 2 strains isolated in 2004 and 2005, and the latter strains are sufficiently similar to the 2004 strain from patient 1 to indicate that transmission had occurred. The map of SNP accumulation for patient 1 (Figure 3*A*) suggests that transmission was associated with clinic visits that occurred in early 2004.

It is notable that all but 2 of the isolates from UK PAD patients and 2 from France and Tunisia, although distinct, are closely related. The other isolates are more diverse but part of the same species. More highly divergent strains may form distinct lineages or may ultimately be described as distinct taxa, but deeper understanding of the population genetics of this organism will only be achieved when more strains are sequenced.

PAD patients received multiple antibiotic treatment courses (Figure 1 and Supplementary Figure 1) and received immunoglobulin regularly. Changing bacterial load may be related to treatment, but the incomplete record makes the relationship between treatment and bacterial load uncertain. We detected resistance mutations in the QRDR of *parC* of all the UK isolates, indicating that these strains are likely to be resistant. This provides genetic evidence for the previously reported clinical finding in patient 1, who was treated with courses of antibiotics, including quinolones, without improvement and who only responded to the pleuromutilin agent valnemulin (Econor) [18]. The substitution of the serine residue at position 89 of the QRDR (80 by *E. coli* numbering) [32] is known to confer quinolone resistance in *M. hominis* [33]. Mutations associated with macrolide resistance were also detected in the PAD patients; later isolates from patient 2 had an A2059G substitution, which is a hotspot mutation conferring macrolide resistance [34].

In summary, we have used genomics to differentiate between persistence and reinfection, providing evidence that *M. amphoriforme* infects immunocompetent and immunocompromised patients [3, 18], is chronic in patients with PAD, and is associated with obstructive airway disease. Transmission can occur in a clinical environment, suggesting that respiratory precautions may be required. The use of next-generation sequencing has allowed us to improve our understanding of the biology and epidemiology of this more rapidly than by phenotypic or other genotyping methods.

## Supplementary Data

Supplementary materials are available at *Clinical Infectious Diseases* online (http://cid.oxfordjournals.org). Supplementary materials consist of data provided by the author that are published to benefit the reader. The posted materials are not copyedited. The contents of all supplementary data are the sole responsibility of the authors. Questions or messages regarding errors should be addressed to the author.

Supplementary Data
