# Supplementary material for: Genomic Investigations Unmask Mycoplasma amphoriforme, a New Respiratory Pathogen
Source: Clin Infect Dis. 2014 Oct 23;60(3):381–8. doi: 10.1093/cid/ciu820 (PMC4293396; doi:10.1093/cid/ciu820)
Supplement: Supplementary Data [file supp_60_3_381__index.html]

Genomic Investigations Unmask Mycoplasma amphoriforme, a New Respiratory Pathogen — Genomic Investigations Unmask Mycoplasma amphoriforme, a New Respiratory Pathogen — Supplementary Data 

# Genomic Investigations Unmask *Mycoplasma amphoriforme*, a New Respiratory Pathogen

## Supplementary Data

Supplementary Data

**Files in this Data Supplement:**

- Supplementary Data - Docx file
