# Supplementary material for: Genomic Investigations Unmask Mycoplasma amphoriforme, a New Respiratory Pathogen
Source: Clin Infect Dis. 2014 Oct 23;60(3):381–8. doi: 10.1093/cid/ciu820 (PMC4293396; doi:10.1093/cid/ciu820)

Supplementary Material

**Supplementary table 1.** Summary of the characteristics of patients providing isolates for whole genome sequencing. All patients were receiving parenteral immunoglobulin replacement therapy. M: male, F: female, XLA: + positive, – negative, NA not applicable ND no data, X-linked antibody deficiency, CVID: common variable immunodeficiency. The duration of observation reflects the number of days between the first and last samples included in the study.

| **Patient number** | **Sex** | **Age** | **Diagnosis** | **Duration of observation** | **Bronchiectasis** | **Airways obstruction** | **Steroids** | **Inhaled bronchodilators** |
| --- | --- | --- | --- | --- | --- | --- | --- | --- |
| 1 | M | 33 | XLA | 1627 | + | - | + | - |
| 2 | F | 32 | CVID | 1542 | + | + | + | + |
| 3 | M | 21 | XLA | 729 | + | - | ND | ND |
| 8 | M | 33 | CVID | 1595 | + | - | - | - |
| 9 | M | 41 | CVID | 1554 | + | - | + | + |
| 25 | M | 33 | CVID | 786 | - | + | + | ND |
| 65 | M | 41 | XLA | 1228 | + | + | + | + |
| 125 | M | 24 | XLA | 1 | - | - | - | - |
| 144 | M | 41 | XLA | 197 | - | + | - | - |
| 13 | M | 2 | beta thalassaemia | NA | - | - | - | ND |
| 14 | F |  | Cystic fibrosis | NA | - | + | + | + |
| 15 | - | 35 | Severe sepsis, interstitial syndrome | NA | - | + | + | ND |

Supplementary Figure S1 Natural history of patients 2, 3 and 8 with *M. amphoriforme* infection. Colony forming units are estimated using the *udg* qPCR. The symbols represent the bacteria isolated and the antibiotic treatment used as follows:


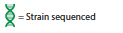

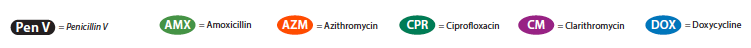


an asterisk indicates that udg PCR was not performed but that the patient was positive by culture or 16S PCR. All patients were receiving regular parenteral immunoglobulin therapy at 2-4 weekly intervals.
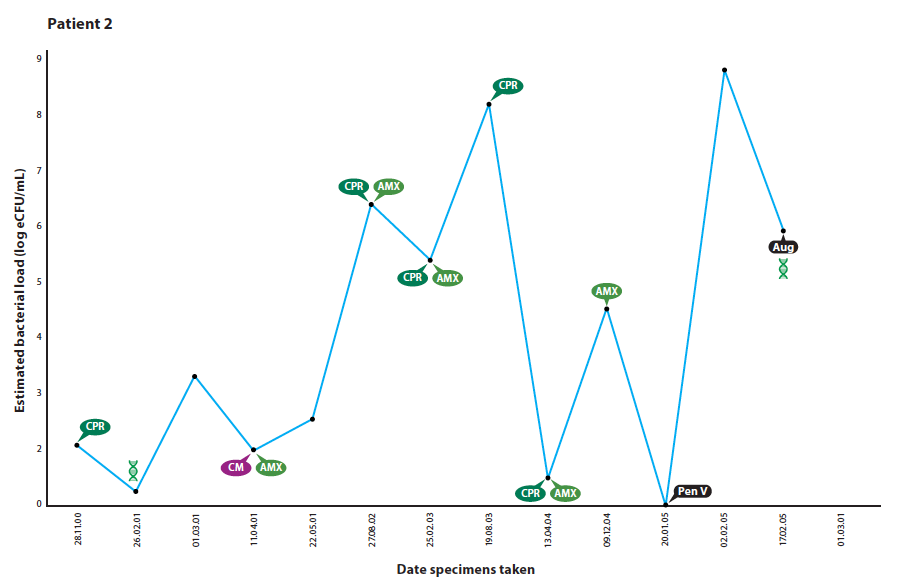


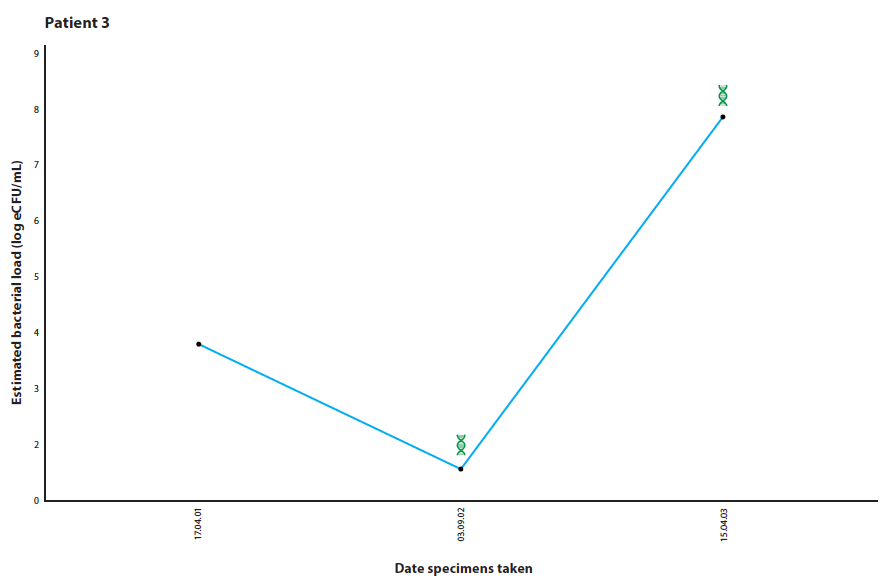


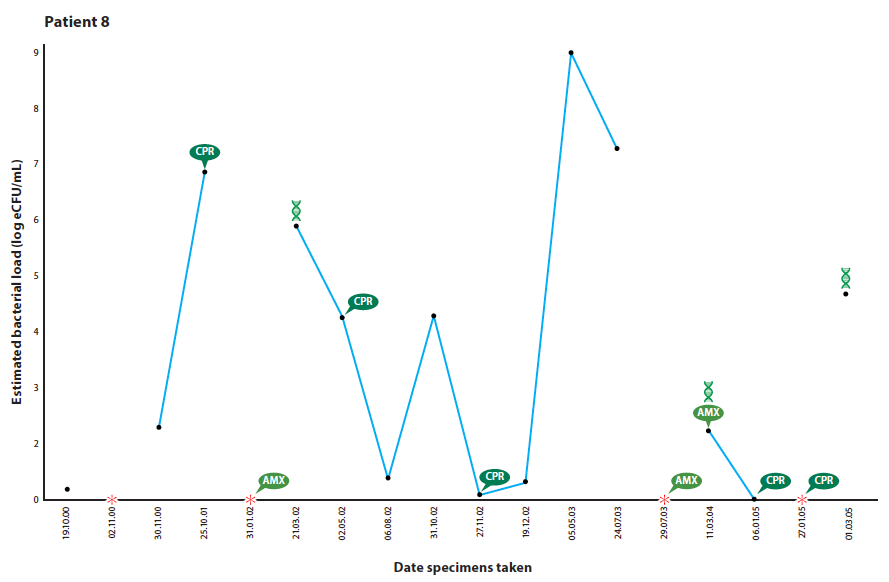


Supplementary Figure 2 Phylogenetic tree for isolates of *M. amphoriforme* distinguished by colour and the strain designated by its code (eight patients from RFL and one of three French/Tunisian isolates) constructed with Gubbins (https://github.com/**sanger**-pathogens/**gubbins)**. A star indicates phylogenetic relationships that are supported by 100 bootstrap analyses scale bar represents number of SNPs, and the 96 and 99 represent the bootstrap support.
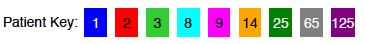


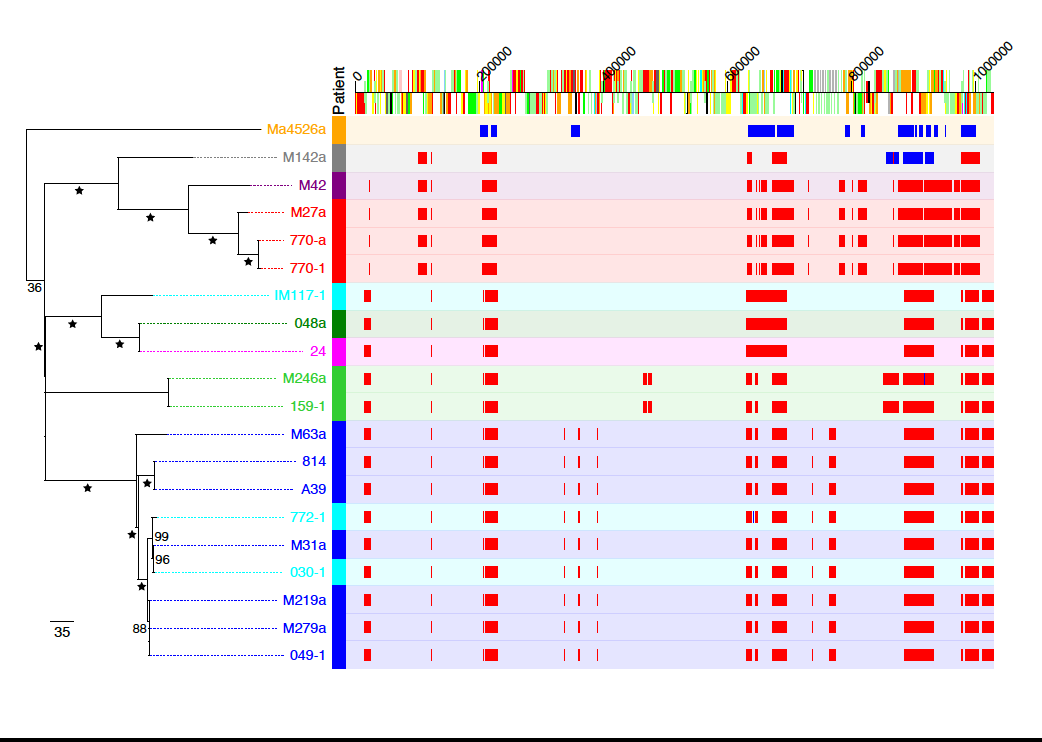


Supplementary figure 3 Single nucleotide polymorphism map of *M. amphoriforme* isolates isolated from nine UK, three French/Tunisianpatients using an in house script. Brown lines indicate SNPs with allele frequency >0.90, Blue frequency <0.90. Black dots indicate zero coverage at that point.


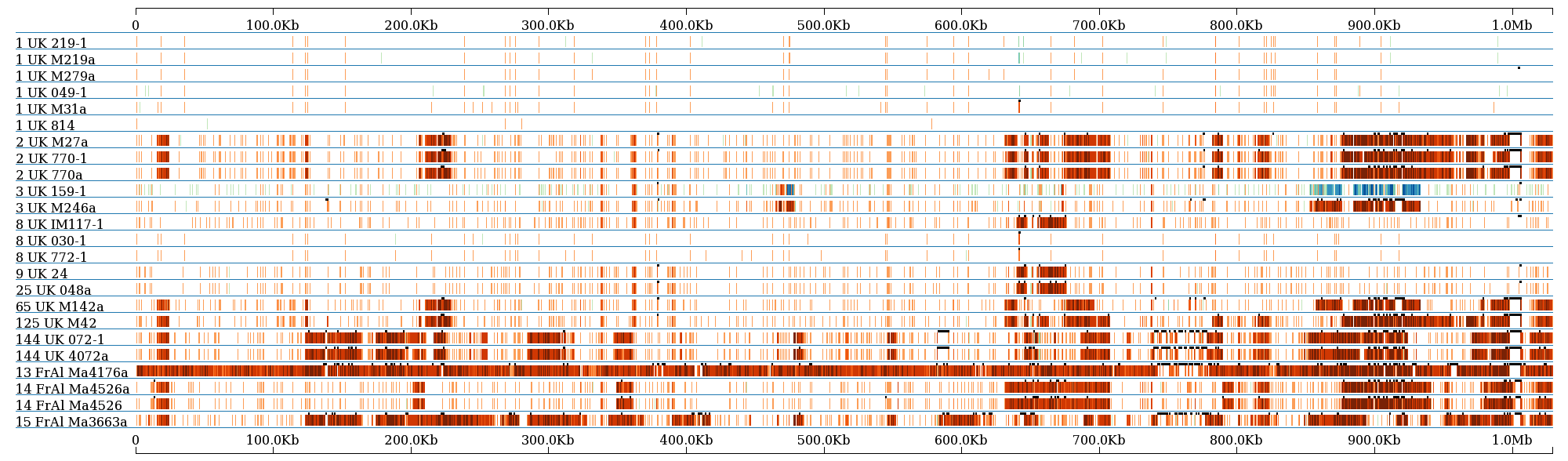

Supplement: Supplementary Data [file supp_ciu820_ciu820supp.docx]
